# Supplementary material for: Study protocol for COVID-RV: a multicentre prospective observational cohort study of right ventricular dysfunction in ventilated patients with COVID-19
Source: BMJ Open. 2021 Jan 13;11(1):e042098. doi: 10.1136/bmjopen-2020-042098 (PMC7811959; doi:10.1136/bmjopen-2020-042098)
Supplement: Author's manuscript [file bmjopen-2020-042098.draft_revisions.pdf]

BMJ Open is committed to open peer review. As part of this commitment we make the peer review history of every article we publish publicly available.

When an article is published we post the peer reviewers' comments and the authors' responses online. We also post the versions of the paper that were used during peer review. These are the versions that the peer review comments apply to.

The versions of the paper that follow are the versions that were submitted during the peer review process. They are not the versions of record or the final published versions. They should not be cited or distributed as the published version of this manuscript.

BMJ Open is an open access journal and the full, final, typeset and author-corrected version of record of the manuscript is available on our site with no access controls, subscription charges or pay-per-view fees (http://bmjopen.bmj.com).

If you have any questions on BMJ Open's open peer review process please email info.bmjopen@bmj.com

BMJ Open

Study protocol for COVID-RV: a multi-centre prospective observational cohort study of right ventricular dysfunction in ventilated patients with COVID-19.

Journal:	*BMJ Open*
Manuscript ID	bmjopen-2020-042098
Article Type:	Protocol
Date Submitted by the Author:	25-Jun-2020
Complete List of Authors:	Willder, Jennifer; NHS Education for Scotland West Region,<br>McCall, Philip; University of Glasgow, Academic unit of Anaesthesia, Pain and Critical Care Medicine; NHS Education for Scotland West Region<br>Messow, Martina ; University of Glasgow, Robertson Centre for Biostatistics<br>Gillies , Mike; The University of Edinburgh, Anaesthesia, Care and PAin Medicine; Edinburgh Royal Infirmary, Department of Anaesthesia<br>Berry, Colin; University of Glasgow, British Heart Foundation Glasgow Cardiovascular Research Centre; Golden Jubilee Hospital, Department of Cardiology<br>Shelley, Benjamin; University of Glasgow, Academic unit of Anaesthesia, Pain and Critical Care Medicine; Golden Jubilee Hospital, Department of Anaesthesia
Keywords:	Adult intensive & critical care < ANAESTHETICS, Adult cardiology < CARDIOLOGY, Echocardiography < CARDIOLOGY, Heart failure < CARDIOLOGY, Respiratory infections < THORACIC MEDICINE

SCHOLARONE™
Manuscripts

I, the Submitting Author has the right to grant and does grant on behalf of all authors of the Work (as defined in the below author licence), an exclusive licence and/or a non-exclusive licence for contributions from authors who are: i) UK Crown employees; ii) where BMJ has agreed a CC-BY licence shall apply, and/or iii) in accordance with the terms applicable for US Federal Government officers or employees acting as part of their official duties; on a worldwide, perpetual, irrevocable, royalty-free basis to BMJ Publishing Group Ltd ("BMJ") its licensees and where the relevant Journal is co-owned by BMJ to the co-owners of the Journal, to publish the Work in this journal and any other BMJ products and to exploit all rights, as set out in our licence.

The Submitting Author accepts and understands that any supply made under these terms is made by BMJ to the Submitting Author unless you are acting as an employee on behalf of your employer or a postgraduate student of an affiliated institution which is paying any applicable article publishing charge ("APC") for Open Access articles. Where the Submitting Author wishes to make the Work available on an Open Access basis (and intends to pay the relevant APC), the terms of reuse of such Open Access shall be governed by a Creative Commons licence – details of these licences and which Creative Commons licence will apply to this Work are set out in our licence referred to above.

Other than as permitted in any relevant BMJ Author's Self Archiving Policies, I confirm this Work has not been accepted for publication elsewhere, is not being considered for publication elsewhere and does not duplicate material already published. I confirm all authors consent to publication of this Work and authorise the granting of this licence.

For peer review only

Title

Study protocol for COVID-RV: a multi-centre prospective observational cohort study of right ventricular dysfunction in ventilated patients with COVID-19.

Authors

Dr Jennifer Mary Willder[1]

Dr Philip McCall[1,2]

Dr Claudia-Martina Messow[3]

Professor Mike Gillies[4,5]

Professor Colin Berry[6,7]

Dr Benjamin Shelley*[2,8]

*corresponding author: postal address: Department of Anaesthesia, Golden Jubilee National Hospital, Agamemnon St, Clydebank G81 4DY. email: Benjamin.Shelley@glasgow.ac.uk.

1. West of Scotland School of Anaesthesia, NHS Education for Scotland, Glasgow, UK.
2. School of Medicine, Dentistry and Nursing, University of Glasgow, Glasgow, UK.
3. Robertson Centre for Biostatistics, Institute of Health and Wellbeing, University of Glasgow, Glasgow, UK.
4. Anaesthesia, Critical Care and Pain Medicine, Division of Health Sciences, University of Edinburgh, Edinburgh, UK.
5. Department of Anaesthesia, Edinburgh Royal Infirmary, Edinburgh, UK.
6. Department of Cardiology, Golden Jubilee National Hospital, Clydebank, UK.
7. Institute of Cardiovascular and Medical Sciences, University of Glasgow, Glasgow, UK.
8. Department of Anaesthesia, Golden Jubilee National Hospital, Clydebank, UK.

Word Count

2236

For peer review only

Keywords

Adult intensive & critical care

Adult cardiology

Echocardiography

Heart failure

Respiratory infections.

For peer review only

ABSTRACT

Introduction

Coronavirus disease 2019 [COVID-19] can cause severe acute respiratory failure requiring management in intensive care (ICU) with invasive ventilation and a 40% mortality rate. Cardiovascular manifestations are common and studies have shown an increase in right ventricular (RV) dysfunction associated with mortality. These studies however, comprise of heterogeneous patient groups with few requiring invasive ventilation. This study will investigate the prevalence and prognostic significance of RV dysfunction in ventilated patients with COVID-19 which may lead to targeted interventions to improve patient outcomes.

Methods and analysis

This prospective multicenter observational cohort study will perform transthoracic echocardiography (TTE) in 150 COVID-19 patients requiring invasive ventilation for more than 48 hours. RV dysfunction will be defined as TTE evidence of RV dilatation along with the presence of septal flattening. Baseline demographics, disease severity data and clinical information relating to proposed aetiological mechanisms of RV dysfunction (acute respiratory distress syndrome (ARDS), disordered coagulation, direct myocardial injury and ventilation) will be collected and analysed.

Primary outcome measures include the prevalence of RV dysfunction and its association with 30-day mortality. Exploratory outcome measures will investigate the association of the proposed aetiological mechanisms of RV dysfunction to the primary outcomes.

Prevalence of RV dysfunction will be determined along with 95% Clopper-Pearson confidence intervals and 30-day survival will be analysed using logistic regression adjusting for patient demographics, phase of disease and baseline severity of illness. The role of potential aetiological factors (ARDS, disordered coagulation, direct myocardial injury and ventilation) in relation to the primary outcomes will be analysed using logistic regression.

Ethics and dissemination

Approval was gained from Scotland A Research Ethics Committee (REC reference 20/SS/0059). Findings will be disseminated by various methods including webinars, international presentations and publication in peer-reviewed journals.

For peer review only

ARTICLE SUMMARY

Strengths and limitations of this study

- Includes the most severely ill patients with COVID-19 disease; a patient group which are under-represented in other studies of RV function in patients with COVID-19.
- Pragmatic study and primary outcome, reflecting clinical practice of ICU echocardiography.
- Appropriately statistically powered.
- RV function is dynamic.
- As with all COVID research, recruitment of adequate participant numbers may be hampered due to the 'passing of the peak' of the COVID-19 pandemic in the UK but new cases continue to emerge.

For peer review only

INTRODUCTION

In December 2019 the first case of a novel coronavirus disease-19 (COVID-19) caused by the SARS-Cov-2 virus was identified in Wuhan, China.[1] Although the majority of cases are mild, approximately 5% of confirmed cases will require intensive care unit (ICU) admission, with severe acute respiratory failure (SARF) being the prominent feature.[2]As of 22nd June 2020 there were over 8.8 million cases worldwide with over 450, 000 confirmed deaths.[3] On the 19th of June 2020 over 13,000 patients had been treated in UK critical care units.[4 5] Unfortunately, in line with other countries, recent UK figures have demonstrated extremely poor ICU outcomes with mortality rates of over 40%.[4]

Although not always classical in presentation, the acute respiratory distress syndrome (ARDS) is widely accepted to be the usual cause of SARF in COVID-19 patients.[6] Along with respiratory symptoms, cardiovascular manifestations are also common and may adversely impact prognosis.[7-9] In ARDS, the more susceptible right ventricle (RV) is subject to impaired function due to increased afterload.[10 11] RV dysfunction is known to be an independent predictor of mortality in the non-COVID-19 setting and increasing ARDS severity is associated with increased frequency of RV dysfunction.[12-15]

Recent studies have shown that RV dysfunction is a common finding in COVID-19 patients and it increases mortality.[9 16 17] Whilst no single causative factor has been elucidated, there are several potential aetiological factors which have been shown to increase RV afterload and have deleterious effects on RV function including ARDS,[10 18 19] disordered coagulation,[20-24] direct myocardial injury,[7 25 26] and positive pressure ventilation with positive end expiratory pressure (PEEP).[18 27] Previous studies addressing RV dysfunction in patients with COVID-19 included heterogeneous groups of patients of varying disease severity, of whom only a small proportion required mechanical ventilation. No adequately powered study has prospectively explored the incidence of RV dysfunction and its association with mortality in ventilated patients with COVID-19. Further investigation and exploration of causative mechanisms may allow targeted interventions to improve outcomes for this patient group.[12 19 28 29]

Hypothesis

Right ventricular failure is common in patients ventilated with COVID-19 and is associated with increased mortality. In examining this hypothesis, we will explore potential mechanisms for RV dysfunction in this patient group.

For peer review only

METHODS AND ANALYSIS

Summary: A multicentre prospective observational study in ventilated patients with COVID-19 in participating Scottish intensive care units.

Centre(s): Fifteen participating ICUs in Scotland.

Study status: This is a planned study that was granted ethical approval on 05/06/2020 and grant funding on 27/05/2020. Recruitment is expected to commence in June 2020.

Selection of study subjects

Inclusion criteria

Patients >16 years with confirmed SARS-CoV-2 infection with SARF requiring tracheal intubation and positive pressure ventilation in intensive care for more than 48 hours, but not more than 14 days will be eligible for inclusion in the study.

Exclusion criteria

Patients for whom informed consent is unable to be provided either directly or on their behalf by a relative/welfare guardian will be excluded. Other exclusion criteria include:

- Pregnancy
- Ongoing participation in any investigational research that may undermine the scientific basis of the study
- Prior participation in the COVID-RV study
- Requirement for extra-corporeal membrane oxygenation (ECMO) support for respiratory or cardiovascular failure
- Patient at end of life and not expected to survive longer than 24 hours

Study Conduct

Recruitment

Potential participants will be identified and recruited by the local treating clinical team during their admission to intensive care.

Consent

For peer review only

Prospective informed consent will be obtained. As target patients will be critically ill in the ICU, it is unlikely that they themselves will be able to provide informed consent. Consent will be sought from the patient's legally designated representative, most likely a relative, either in person or via telephone. Further consent will then be sought from the study participant if they recover sufficiently and regain capacity to provide consent.

Medical management

Medical management will be according to the standard of care at each treating site and is not influenced by this study protocol.

Study interventions

Echocardiography conduct

Participants recruited to take part in the study will undergo a single transthoracic echocardiogram (TTE) to determine the presence or absence of RV dysfunction. This will be undertaken after 48 hours but before 14 days of intubation and invasive ventilation. TTE will be performed by a range of appropriately competent practitioners including (but not exclusively); intensive care clinicians, cardiologists and specialist echocardiographers reflecting the clinical practice of bedside echocardiography in intensive care. For the purposes of the primary outcome, imaging required will be in keeping with the protocol required for a Focused Intensive Care Echo (FICE) scan.[30] A secondary quantitative dataset will be collected where echocardiography skills allow.

Laboratory sampling

High sensitivity troponin (I or T) and where available natriuretic peptides (BNP or NT-proBNP) will be measured as part of routine blood sampling on the same day as TTE.

Data collection and management

Data collection will be performed by the local study team on case report forms (CRFs) which will be filed and securely stored at participating sites. The data will be anonymised at site and a unique alphanumeric study number allocated. Completed CRFs will then be entered onto a secure online database in a linked-anonymised form. Electronic data will be stored in an encrypted and anonymised format for ten years following the completion of the trial. At the end of this period, the dataset will be destroyed and this will be performed to DoD 5220.22-M standards. The study will adhere to all data protection regulations and all data will be held in accordance with The General Data Protection Regulation (2018).

For peer review only

Clinical data

Clinical data involves baseline demographic data and chronic comorbidities, ICU admission information, severity of illness and acute comorbidities and follow-up data. Clinical data relating to the proposed mechanisms of RV dysfunction will also be collected, specifically regarding ARDS, disordered coagulation, myocardial injury and ventilation.

Laboratory data

Laboratory data will be obtained from the local biochemistry and haematology laboratory reporting systems on the day of echocardiography and at follow-up.

ECG data

A 12 lead ECG will be anonymised and transferred electronically to the research team for further analysis. Abnormalities in rate, rhythm, conduction and repolarisation (ST-segment deviation, T-wave inversion) will be classified, blind to other clinical data.

Echocardiography data

A focused echocardiography dataset will be used to answer the primary outcome (Table 1). If available, and the echocardiographer's competency and experience permit, further quantitative measures of RV function will be obtained at this time (Table 2).

Table 1. Focused echocardiography dataset relating to primary outcome

**ECHOCARDIOGRAPHY PARAMETER**	**RESULT**	
**RV Dilatation**	Yes	
	No	
	Unable to assess	
**Septal flattening**	Yes	Systole
		Diastole
		Both
	No	
	Unable to assess	
**RV Function**	Normal	
	RV Dysfunction	
	Unable to assess	
**Free text:** *Comments*		
**LV function**	Normal	
	LV Dysfunction	

For peer review only

	Unable to assess
**LV Dilatation**	Normal
	LV Dilatation
	Unable to assess
**Free text:** *Comments.*	

Table 1 Legend Basic echocardiography dataset to allow determination of the primary outcome measures.

As part of the dataset, a parasternal short axis (PSAX) and apical 4-chamber (A4C) 'loop' will be recorded and saved. For the A4C loop this will take the form of an RV focused view. For both, the images will be optimised, have ECG monitoring present throughout and the frame rate should be maximised; both will be recorded for 4 beats.[31] This data will be anonymised and transferred electronically to the research team for further analysis.

Table 2. Detailed echocardiography measures of RV function

**ECHOCARDIOGRAPHY PARAMETER**		**RESULT**
**RV area**	**RV end diastolic area**	cm^2
	**RV end systolic area**	cm^2
	**Fractional area change (FAC).**	%
		Unable to assess
**Free text:** *Image quality, RWMA, McConnell's sign, presence of clot etc.*		
**Triscupid annular plane systolic excursion (TAPSE)**		mm
		Unable to assess
**S' wave velocity at tricuspid annulus (S'Wave)**		cm/s
		Unable to assess
**RV dimensions**	**RVD1**	mm
	**RVD2**	mm
	**RVD3**	mm
		Unable to assess
**TR V_{max}** (to allow calculation of PA systolic pressure)		m/s
		Unable to assess
**Tricuspid regurgitation**		None
		Mild
		Moderate
		Severe
		Unable to assess
**Free text:** *Method of assessing TR (vena contracta, quantitative [EROA, ERV], hepatic vein flow*		

For peer review only

**Right ventricular index of myocardial performance (RIMP) / Tei Index**	**PWD**		
	**Tissue Doppler**		
			Unable to assess
**IVC diameter**	**IVC Diameter**		cm
	**Resp variation /collapse >50%**		Yes
			No
			Unable to assess
**RVOT Acceleration Time**			ms
			Unable to assess
**LV function and size**	**2D**	**LVIDd**	cm
		**LVIDs**	cm
		**LVFS**	%
			Unable to assess
	**Bi-Plane**	**LVEDV**	ml
		**LVESV**	ml
		**LVEF**	%
			Unable to assess
**Free text:** *Comments such as regional wall motion abnormality, valve pathology, etc etc.*			

Table 2. Legend Detailed echocardiography measures of RV function to be undertaken dependent upon the echocardiographer's competency and experience and if image quality allows.

Study Outcomes

Primary outcome

The primary outcome of the study is the prevalence of RV dysfunction in ventilated patients with COVID-19 and its association with 30-day mortality. RV dysfunction will be defined as TTE evidence of RV dilatation along with the presence of septal flattening (in systole, diastole or both).

Justification for primary outcome

TTE is the mainstay of RV imaging in ICU; it is non-invasive, free from ionising radiation widely available and low cost. The ability to bring the echo machine to the patient bedside means it is the preferred method for diagnosis of RV dysfunction in ICU patients.[32] TTE can determine RV dilatation and can detect eccentric movement of the interventricular septum. The combination of RV dilatation and eccentric septal motion is often referred to as acute cor pulmonale and is a widely reported measure of RV dysfunction in patients with respiratory failure in intensive care.[13 14 33-35]

For peer review only

For peer review only - http://bmjopen.bmj.com/site/about/guidelines.xhtml

Exploratory outcomes

Exploratory outcome measures largely relate to the association of the proposed aetiological factors, namely ARDS, disordered coagulation, direct myocardial injury and ventilation, to RV dysfunction and 30 day mortality. The association of cardiac biomarkers (troponin T or I and natriuretic peptides (BNP or NT-proBNP)) to the primary outcome measures will also be evaluated.

Saved echo-loops will be analysed centrally at the host institution, blinded and anonymised under the supervision of BSE accredited echocardiographers. 2D speckle tracking will be performed for analysis for RV free-wall and global longitudinal strain.; image analysis will be performed on the TOMTEC 2D-Cardiac Performance Analysis platform.

Statistical considerations

All statistical analyses will be performed in conjunction with the Robertson Centre for Biostatistics at the University of Glasgow.

Sample size calculation

Sample size selection is required to be a pragmatic balance of maximising available information versus the prompt delivery of the study and reporting of results to the clinical workforce to inform care. We believe it is realistic to recruit 120-150 patients across participating sites.

Prevalence of RV dysfunction: with a sample size of 120, if the proportion of patients with RV dysfunction was 50%, the 95% confidence interval around the estimated rate would be 40.7% to 59.3%, if the proportion was 25% the 95% confidence interval would be 17.5% to 33.7%. With a sample size of 150 patients, the 95% confidence intervals would be (41.7%, 58.3%) and (18.6%, 33.1%) respectively.

Association with mortality: Table 1 demonstrates the differences in mortality rates that could be detected in a sample size of 120-150 patients at a significance level of 5% and proportions of patients with RV dysfunction of 25% and 50%, assuming an overall mortality rate of 50% (as presented in the recent UK ICNARC report on COVID-19 in critical care).[36]

Table 3. Indicative Power

For peer review only

Sample Size	Power	Number *without* RV dysfunction	Number *with* RV dysfunction	Mortality in patients *without* RV dysfunction	Mortality in patients *with* RV dysfunction	Odds Ratio
120	80%	60	60	0.373	0.627	2.83
120	90%	60	60	0.354	0.646	3.33
150	80%	75	75	0.386	0.614	2.53
150	90%	75	75	0.369	0.631	2.92
120	80%	90	30	0.427	0.719	3.43
120	90%	90	30	0.417	0.749	4.17
150	80%	112	38	0.435	0.695	2.96
150	90%	112	38	0.426	0.722	3.50

Table 3. Legend Differences in mortality rates that could be detected at a 5% significance level for sample sizes of 120 and 150, and proportions of patients with RV dysfunction of 25% and 50%, assuming an overall mortality rate of 50%.

Data analysis

Primary outcome

The proportion of ventilated patients with COVID-19 who have RV dysfunction will be determined, with a 95% confidence interval (Clopper-Pearson method). The association of RV dysfunction with 30-day mortality will then be analysed using logistic regression analysis predicting 30-day ICU mortality from presence or absence of RV dysfunction, adjusting for patient demographics, phase of disease and baseline severity of illness.

Exploratory outcomes

To assess the role of potential aetiological factors (ARDS, disordered coagulation, direct myocardial injury and ventilation) for RV dysfunction, the relation of each measure of the aetiological factors to the outcomes will be analysed in the same way as for the primary outcome. A more in-depth analysis will include carrying out a principal component analysis for all measures within each factor, in order to create one single measure (the first principal component) representing each factor. The relation of these to the outcomes will be assessed in regression analyses analogously to the ones described above. This will be done for each factor separately and also in combination. Other approaches, e.g. the lasso, for determining the role of the individual measures of all factors may be explored.

For peer review only

The association of cardiac biomarkers with RV dysfunction will be analysed using logistic regression analysis predicting presence or absence of RV dysfunction from the biomarker level, adjusting for patient demographics, phase of disease and baseline severity. Association of the biomarkers with 30-day mortality will be analysed in a similar manner. To assess whether any association differs in the presence or absence of RV dysfunction, RV dysfunction and its interaction with the biomarker will be added to the model.

Patient and public involvement statement

Due to the nature of the current COVID-19 pandemic and the subsequent urgency to establish this study there was no patient or public involvement. Patients were not invited to comment on the study design and were not consulted to develop patient relevant outcomes or interpret the results. Patients were not invited to contribute to the writing or editing of this document for readability or accuracy.

For peer review only

ETHICS AND DISSEMINATION

Ethical approval was granted by the Scotland A Research Ethics Committee (REC reference 20/SS/0059) on 05/06/2020.

The safety profile of all research interventions; TTE, collection of clinical information and blood specimens, is well established and therefore adverse event reporting is not applicable.

Given the nature of the current global pandemic we will endeavor to publish the prevalence of RV dysfunction in COVID-19 as soon as possible on completion of the study. Once the thirty-day follow-up period is complete, the primary outcome will be published along with all available exploratory analyses. We envisage sharing findings with the critical care community at an early stage through a series of webinars.

For peer review only

REFERENCES

1. Zhu N, Zhang D, Wang W, et al. A Novel Coronavirus from Patients with Pneumonia in China, 2019. The New England journal of medicine 2020;382(8):727-33 doi: 10.1056/NEJMoa2001017[published Online First: Epub Date]|.

2. Wu Z, McGoogan JM. Characteristics of and Important Lessons From the Coronavirus Disease 2019 (COVID-19) Outbreak in China: Summary of a Report of 72314 Cases From the Chinese Center for Disease Control and Prevention. Jama 2020 doi: 10.1001/jama.2020.2648[published Online First: Epub Date]|.

3. World Health Organisation. Coronavirus disease (COVID-19) Situation Report – 154. In: WHO, ed., 2020.

4. Intensive Care National Audit & Research Centre (ICNARC). ICNARC report on COVID-19 in critical care 19 June 2020 London: ICNARC, 2020.

5. Scottish Intensive Care Society Audit Group (SICSAG). Scottish Intensive Care Society Audit Group report on COVID-19. Edinburgh: Public Health Scotland, 2020.

6. Gattinoni L, Chiumello D, Rossi S. COVID-19 pneumonia: ARDS or not? Critical Care 2020;24(1):154 doi: 10.1186/s13054-020-02880-z[published Online First: Epub Date]|.

7. Fried JA, Ramasubbu K, Bhatt R, et al. The Variety of Cardiovascular Presentations of COVID-19. Circulation;0(0) doi: doi:10.1161/CIRCULATIONAHA.120.047164[published Online First: Epub Date]|.

8. Ruan Q, Yang K, Wang W, et al. Clinical predictors of mortality due to COVID-19 based on an analysis of data of 150 patients from Wuhan, China. Intensive Care Med 2020 doi: 10.1007/s00134-020-05991-x[published Online First: Epub Date]|.

9. Szekely Y, Lichter Y, Taieb P, et al. The Spectrum of Cardiac Manifestations in Coronavirus Disease 2019 (COVID-19) - a Systematic Echocardiographic Study. Circulation 2020 doi: 10.1161/CIRCULATIONAHA.120.047971[published Online First: Epub Date]|.

10. Lahm T, Douglas IS, Archer SL, et al. Assessment of Right Ventricular Function in the Research Setting: Knowledge Gaps and Pathways Forward. An Official American Thoracic Society Research Statement. Am J Respir Crit Care Med 2018;198(4):e15-e43 doi: 10.1164/rccm.201806-1160ST[published Online First: Epub Date]|.

For peer review only

11. Grignola JC, Domingo E. Acute Right Ventricular Dysfunction in Intensive Care Unit. BioMed research international 2017;2017:8217105 doi: 10.1155/2017/8217105[published Online First: Epub Date]|.

12. Zochios V, Parhar K, Vieillard-Baron A. Protecting the Right Ventricle in ARDS: The Role of Prone Ventilation. Journal of Cardiothoracic and Vascular Anesthesia 2018;32(5):2248-51 doi: https://doi.org/10.1053/j.jvca.2018.01.007[published Online First: Epub Date]|.

13. Boissier F, Katsahian S, Razazi K, et al. Prevalence and prognosis of cor pulmonale during protective ventilation for acute respiratory distress syndrome. Intensive care medicine 2013;39(10):1725-33 doi: 10.1007/s00134-013-2941-9[published Online First: Epub Date]|.

14. Mekontso Dessap A, Boissier F, Charron C, et al. Acute cor pulmonale during protective ventilation for acute respiratory distress syndrome: prevalence, predictors, and clinical impact. Intensive Care Med 2016;42(5):862-70 doi: 10.1007/s00134-015-4141-2[published Online First: Epub Date]|.

15. Shah TG, Wadia SK, Kovach J, et al. Echocardiographic parameters of right ventricular function predict mortality in acute respiratory distress syndrome: a pilot study. Pulm. Circ. 2016;6(2):155-60 doi: 10.1086/685549[published Online First: Epub Date]|.

16. Li Y, Li H, Zhu S, et al. Prognostic Value of Right Ventricular Longitudinal Strain in Patients with COVID-19. JACC: Cardiovascular Imaging 2020:3423 doi: 10.1016/j.jcmg.2020.04.014[published Online First: Epub Date]|.

17. Argulian E, Sud K, Vogel B, et al. Right Ventricular Dilation in Hospitalized Patients with COVID-19 Infection. JACC. Cardiovascular imaging 2020 doi: 10.1016/j.jcmg.2020.05.010[published Online First: Epub Date]|.

18. Vieillard-Baron A, Matthay M, Teboul JL, et al. Experts' opinion on management of hemodynamics in ARDS patients: focus on the effects of mechanical ventilation. Intensive care medicine 2016;42(5):739-49 doi: 10.1007/s00134-016-4326-3[published Online First: Epub Date]|.

19. Paternot A, Repesse X, Vieillard-Baron A. Rationale and Description of Right Ventricle-Protective Ventilation in ARDS. Respiratory care 2016;61(10):1391-6 doi: 10.4187/respcare.04943[published Online First: Epub Date]|.

20. British Thoracic S. BTS Guidance on Venous Thromboembolic Disease in patients with COVID-19. Secondary BTS Guidance on Venous Thromboembolic Disease in patients with COVID-19 2020.

For peer review only

file:///C:/Users/mccal/Downloads/BTS%20Guidance%20on%20Venous%20Thromboembolic%20Disease%20in%20patients%20with%20COVID-19.pdf.

21. Zhou F, Yu T, Du R, et al. Clinical course and risk factors for mortality of adult inpatients with COVID-19 in Wuhan, China: a retrospective cohort study. The Lancet 2020;395(10229):1054-62 doi: 10.1016/S0140-6736(20)30566-3[published Online First: Epub Date]|.

22. Liu PP, Blet A, Smyth D, et al. The Science Underlying COVID-19: Implications for the Cardiovascular System. Circulation;0(0) doi: doi:10.1161/CIRCULATIONAHA.120.047549[published Online First: Epub Date]|.

23. Klok FA, Kruip MJHA, van der Meer NJM, et al. Incidence of thrombotic complications in critically ill ICU patients with COVID-19. Thrombosis Research 2020 doi: https://doi.org/10.1016/j.thromres.2020.04.013[published Online First: Epub Date]|.

24. Fox SE, Akmatbekov A, Harbert JL, et al. Pulmonary and Cardiac Pathology in Covid-19: The First Autopsy Series from New Orleans. medRxiv 2020:2020.04.06.20050575 doi: 10.1101/2020.04.06.20050575[published Online First: Epub Date]|.

25. Inciardi RM, Lupi L, Zaccone G, et al. Cardiac Involvement in a Patient With Coronavirus Disease 2019 (COVID-19). JAMA Cardiology 2020 doi: 10.1001/jamacardio.2020.1096[published Online First: Epub Date]|.

26. Augoustides JG. Cardiovascular Consequences and Considerations of Coronavirus Infection – perspectives for the cardiothoracic anesthesiologist and intensivist during the coronavirus crisis. Journal of cardiothoracic and vascular anesthesia 2020 doi: https://doi.org/10.1053/j.jvca.2020.04.001[published Online First: Epub Date]|.

27. Acute Respiratory Distress Syndrome N, Brower RG, Matthay MA, et al. Ventilation with lower tidal volumes as compared with traditional tidal volumes for acute lung injury and the acute respiratory distress syndrome. The New England journal of medicine 2000;342(18):1301-8 doi: 10.1056/NEJM200005043421801[published Online First: Epub Date]|.

28. Murphy E, Shelley B. Clinical presentation and management of right ventricular dysfunction. BJA Education 2019;19(6):183-90 doi: 10.1016/j.bjae.2019.02.004[published Online First: Epub Date]|.

29. Vieillard-Baron A, Charron C, Caille V, et al. Prone positioning unloads the right ventricle in severe ARDS. Chest 2007;132(5):1440-6 doi: 10.1378/chest.07-1013[published Online First: Epub Date]|.

For peer review only

30. Intensive Care Society. FICE Accreditation Pack. Secondary FICE Accreditation Pack 2017.

31. Zaidi A, Knight DS, Augustine DX, et al. Echocardiographic assessment of the right heart in adults: a practical guideline from the British Society of Echocardiography. 2020;7(1):G19 doi: 10.1530/erp-19-0051[published Online First: Epub Date]|.

32. Vieillard-Baron A, Naeije R, Haddad F, et al. Diagnostic workup, etiologies and management of acute right ventricle failure : A state-of-the-art paper. Intensive care medicine 2018;44(6):774-90 doi: 10.1007/s00134-018-5172-2[published Online First: Epub Date]|.

33. Repessé X, Charron C, Vieillard-Baron A. Acute Cor Pulmonale in ARDS: Rationale for Protecting the Right Ventricle. Chest 2015;147(1):259-65 doi: https://doi.org/10.1378/chest.14-0877[published Online First: Epub Date]|.

34. Vieillard-Baron A, Prin S, Chergui K. Echo–Doppler demonstration of acute cor pulmonale at the bedside in the medical intensive care unit. Am J Respir Crit Care Med. 2002;166 doi: 10.1164/rccm.200202-146CC[published Online First: Epub Date]|.

35. Vieillard-Baron A, Schmitt JM, Augarde R. Acute cor pulmonale in acute respiratory distress syndrome submitted to protective ventilation: incidence, clinical implications, and prognosis. Crit Care Med. 2001;29 doi: 10.1097/00003246-200108000-00009[published Online First: Epub Date]|.

36. Intensive Care National Audit & Research Centre (ICNARC). ICNARC report on COVID-19 in critical care. 24th April 2020. Secondary ICNARC report on COVID-19 in critical care. 24th April 2020 2020. https://www.icnarc.org/DataServices/Attachments/Download/c5a62b13-6486-ea11-9125-00505601089b.

For peer review only

AUTHORS' CONTRIBUTIONS

BS and PM conceived of the study and BS is the grant holder. MG and CB assisted in the initial study design. JW wrote the patient documentation and developed the case report forms and online data collection database. C-MM provided guidance on sample size requirements and statistical analysis and will be overseeing the statistical analyses. All authors contributed to refinement of the study protocol and approved the final manuscript.

FUNDING STATEMENT

This work was supported by Medical Research Scotland, grant number CVG-1730-2020. Professor Colin Berry is supported by British Heart Foundation Centre of Research Excellence grant (reference number RE/18/6/34217).

COMPETING INTERESTS STATEMENT

Dr. Shelley reports personal fees from Janssen-Cilag Ltd, outside the submitted work.

For peer review only

BMJ Open

Study protocol for COVID-RV: a multi-centre prospective observational cohort study of right ventricular dysfunction in ventilated patients with COVID-19.

Journal:	*BMJ Open*
Manuscript ID	bmjopen-2020-042098.R1
Article Type:	Protocol
Date Submitted by the Author:	09-Oct-2020
Complete List of Authors:	Willder, Jennifer; NHS Education for Scotland West Region,<br>McCall, Philip; University of Glasgow, Academic unit of Anaesthesia, Pain and Critical Care Medicine; Golden Jubilee Hospital, Department of Cardiology<br>Messow, Martina ; University of Glasgow, Robertson Centre for Biostatistics<br>Gillies , Mike; The University of Edinburgh, Anaesthesia, Care and PAin Medicine; Edinburgh Royal Infirmary, Department of Anaesthesia<br>Berry, Colin; University of Glasgow, British Heart Foundation Glasgow Cardiovascular Research Centre; Golden Jubilee Hospital, Department of Cardiology<br>Shelley, Benjamin; University of Glasgow, Academic unit of Anaesthesia, Pain and Critical Care Medicine; Golden Jubilee Hospital, Department of Anaesthesia
**Primary Subject Heading**:	Intensive care
Secondary Subject Heading:	Cardiovascular medicine, Intensive care, Diagnostics
Keywords:	Adult intensive & critical care < ANAESTHETICS, Adult cardiology < CARDIOLOGY, Echocardiography < CARDIOLOGY, Heart failure < CARDIOLOGY, Respiratory infections < THORACIC MEDICINE

SCHOLARONE™
Manuscripts

I, the Submitting Author has the right to grant and does grant on behalf of all authors of the Work (as defined in the below author licence), an exclusive licence and/or a non-exclusive licence for contributions from authors who are: i) UK Crown employees; ii) where BMJ has agreed a CC-BY licence shall apply, and/or iii) in accordance with the terms applicable for US Federal Government officers or employees acting as part of their official duties; on a worldwide, perpetual, irrevocable, royalty-free basis to BMJ Publishing Group Ltd ("BMJ") its licensees and where the relevant Journal is co-owned by BMJ to the co-owners of the Journal, to publish the Work in this journal and any other BMJ products and to exploit all rights, as set out in our licence.

The Submitting Author accepts and understands that any supply made under these terms is made by BMJ to the Submitting Author unless you are acting as an employee on behalf of your employer or a postgraduate student of an affiliated institution which is paying any applicable article publishing charge ("APC") for Open Access articles. Where the Submitting Author wishes to make the Work available on an Open Access basis (and intends to pay the relevant APC), the terms of reuse of such Open Access shall be governed by a Creative Commons licence – details of these licences and which Creative Commons licence will apply to this Work are set out in our licence referred to above.

Other than as permitted in any relevant BMJ Author's Self Archiving Policies, I confirm this Work has not been accepted for publication elsewhere, is not being considered for publication elsewhere and does not duplicate material already published. I confirm all authors consent to publication of this Work and authorise the granting of this licence.

For peer review only

Title

Study protocol for COVID-RV: a multi-centre prospective observational cohort study of right ventricular dysfunction in ventilated patients with COVID-19.

Authors

Dr Jennifer Mary Willder[1]

Dr Philip McCall[2, 8]

Dr Claudia-Martina Messow[3]

Professor Mike Gillies[4,5]

Professor Colin Berry[6,7]

Dr Benjamin Shelley*[2,8]

*corresponding author: postal address: Department of Anaesthesia, Golden Jubilee National Hospital, Agamemnon St, Clydebank G81 4DY. email: Benjamin.Shelley@glasgow.ac.uk.

1. West of Scotland School of Anaesthesia, NHS Education for Scotland, Glasgow, UK.
2. School of Medicine, Dentistry and Nursing, University of Glasgow, Glasgow, UK.
3. Robertson Centre for Biostatistics, Institute of Health and Wellbeing, University of Glasgow, Glasgow, UK.
4. Anaesthesia, Critical Care and Pain Medicine, Division of Health Sciences, University of Edinburgh, Edinburgh, UK.
5. Department of Anaesthesia, Edinburgh Royal Infirmary, Edinburgh, UK.
6. Department of Cardiology, Golden Jubilee National Hospital, Clydebank, UK.
7. Institute of Cardiovascular and Medical Sciences, University of Glasgow, Glasgow, UK.
8. Department of Anaesthesia, Golden Jubilee National Hospital, Clydebank, UK.

Word Count

2683

For peer review only

Keywords

Adult intensive & critical care

Adult cardiology

Echocardiography

Heart failure

Respiratory infections.

For peer review only

ABSTRACT

Introduction

Coronavirus disease 2019 [COVID-19] can cause severe acute respiratory failure requiring management in intensive care (ICU) with invasive ventilation and a 40% mortality rate. Cardiovascular manifestations are common and studies have shown an increase in right ventricular (RV) dysfunction associated with mortality. These studies however, comprise of heterogeneous patient groups with few requiring invasive ventilation. This study will investigate the prevalence and prognostic significance of RV dysfunction in ventilated patients with COVID-19 which may lead to targeted interventions to improve patient outcomes.

Methods and analysis

This prospective multicenter observational cohort study will perform transthoracic echocardiography (TTE) in 150 COVID-19 patients requiring invasive ventilation for more than 48 hours. RV dysfunction will be defined as TTE evidence of RV dilatation along with the presence of septal flattening. Baseline demographics, disease severity data and clinical information relating to proposed aetiological mechanisms of RV dysfunction (acute respiratory distress syndrome (ARDS), disordered coagulation, direct myocardial injury and ventilation) will be collected and analysed.

Primary outcome measures include the prevalence of RV dysfunction and its association with 30-day mortality. Exploratory outcome measures will investigate the association of the proposed aetiological mechanisms of RV dysfunction to the primary outcomes.

Prevalence of RV dysfunction will be determined along with 95% Clopper-Pearson confidence intervals and 30-day survival will be analysed using logistic regression adjusting for patient demographics, phase of disease and baseline severity of illness. The role of potential aetiological factors (ARDS, disordered coagulation, direct myocardial injury and ventilation) in relation to the primary outcomes will be analysed using logistic regression.

Ethics and dissemination

Approval was gained from Scotland A Research Ethics Committee (REC reference 20/SS/0059). Findings will be disseminated by various methods including webinars, international presentations and publication in peer-reviewed journals.

For peer review only

ARTICLE SUMMARY

Strengths and limitations of this study

- Includes the most severely ill patients with COVID-19 disease; a patient group which are under-represented in other studies of RV function in patients with COVID-19.
- Pragmatic study and primary outcome, reflecting clinical practice of ICU echocardiography.
- Appropriately statistically powered.
- RV function is dynamic.
- As with all COVID research, recruitment of adequate participant numbers may be hampered due to the 'passing of the peak' of the COVID-19 pandemic in the UK but new cases continue to emerge.

For peer review only

INTRODUCTION

In December 2019 the first case of a novel coronavirus disease-19 (COVID-19) caused by the SARS-Cov-2 virus was identified in Wuhan, China.[1] Although the majority of cases are mild, approximately 5% of confirmed cases will require intensive care unit (ICU) admission, with severe acute respiratory failure (SARF) being the prominent feature.[2] As of 27th September 2020 there were over 32.7 million cases worldwide with over 990, 000 confirmed deaths.[3] On the 19th of June 2020 over 11,500 patients had been treated in UK critical care units.[4, 5] Unfortunately, in line with other countries, UK figures have demonstrated extremely poor ICU outcomes with mortality rates of around 40%.[4]

Although not always classical in presentation, the acute respiratory distress syndrome (ARDS) is widely accepted to be the usual cause of SARF in COVID-19 patients.[6] Along with respiratory symptoms, cardiovascular manifestations are also common and may adversely impact prognosis.[7-9] In ARDS, the more susceptible right ventricle (RV) is subject to impaired function due to increased afterload.[10, 11] RV dysfunction is known to be an independent predictor of mortality in the non-COVID-19 setting and increasing ARDS severity is associated with increased frequency of RV dysfunction.[12-15]

Recent studies have shown that RV dysfunction is a common finding in COVID-19 patients and it increases mortality.[9, 16, 17] Whilst no single causative factor has been elucidated, there are several potential aetiological factors which have been shown to increase RV afterload and impair RV contractility, with both factors leading to RV dysfunction. ARDS,[10, 18, 19] pulmonary micro & macro-thrombosis,[20-24] and positive pressure ventilation with positive end expiratory pressure (PEEP)[18, 25] have all been shown to increase RV afterload with RV contractility being depressed by direct myocardial injury.[7, 26, 27] Previous studies addressing RV dysfunction in patients with COVID-19 included heterogeneous groups of patients of varying disease severity, of whom only a small proportion required mechanical ventilation. No adequately powered study has prospectively explored the incidence of RV dysfunction and its association with mortality in ventilated patients with COVID-19. Further investigation and exploration of causative mechanisms may allow targeted interventions to improve outcomes for this patient group.[12, 19, 28, 29]

Hypothesis

Right ventricular failure is common in patients ventilated with COVID-19 and is associated with increased mortality. In examining this hypothesis, we will explore potential mechanisms for RV dysfunction in this patient group.

For peer review only

METHODS AND ANALYSIS

Summary: A multicentre prospective observational study in ventilated patients with COVID-19 in participating Scottish intensive care units.

Centre(s): Fifteen participating ICUs in Scotland.

Study status: This is a planned study that was granted ethical approval on 05/06/2020 and grant funding on 27/05/2020. Recruitment commenced in September 2020.

Selection of study subjects

Inclusion criteria

Patients >16 years with confirmed SARS-CoV-2 infection with SARF requiring tracheal intubation and positive pressure ventilation in intensive care for more than 48 hours, but not more than 14 days will be eligible for inclusion in the study.

Exclusion criteria

Patients for whom informed consent is unable to be provided either directly or on their behalf by a relative/welfare guardian will be excluded. Other exclusion criteria include:

- Pregnancy
- Ongoing participation in any investigational research that may undermine the scientific basis of the study
- Prior participation in the COVID-RV study
- Requirement for extra-corporeal membrane oxygenation (ECMO) support for respiratory or cardiovascular failure
- Patient at end of life and not expected to survive longer than 24 hours

Study Conduct

Recruitment

Potential participants will be identified and recruited by the local treating clinical team during their admission to intensive care.

Consent

For peer review only

Prospective informed consent will be obtained. As target patients will be critically ill in the ICU, it is unlikely that they themselves will be able to provide informed consent. Consent will be sought from the patient's legally designated representative, most likely a relative, either in person or via telephone. Further consent will then be sought from the study participant if they recover sufficiently and regain capacity to provide consent.

Medical management

Medical management will be according to the standard of care at each treating site and is not influenced by this study protocol.

Study interventions

Echocardiography conduct

Participants recruited to take part in the study will undergo a single transthoracic echocardiogram (TTE) to determine the presence or absence of RV dysfunction. This will be undertaken after 48 hours but before 14 days of intubation and invasive ventilation. TTE will be performed by a range of appropriately competent practitioners including (but not exclusively); intensive care clinicians, cardiologists and specialist echocardiographers reflecting the clinical practice of bedside echocardiography in intensive care. For the purposes of the primary outcome, imaging required will be in keeping with the protocol required for a Focused Intensive Care Echo (FICE) scan.[30] A secondary quantitative dataset will be collected where echocardiography skills allow.

Laboratory sampling

High sensitivity troponin (I or T) and where available natriuretic peptides (BNP or NT-proBNP) will be measured as part of routine blood sampling on the same day as TTE.

Data collection and management

Data collection will be performed by the local study team on case report forms (CRFs) which will be filed and securely stored at participating sites. The data will be anonymised at site and a unique alphanumeric study number allocated. Completed CRFs will then be entered onto a secure online database in a linked-anonymised form. Electronic data will be stored in an encrypted and anonymised format for ten years following the completion of the trial. At the end of this period, the dataset will be destroyed and this will be performed to DoD 5220.22-M standards. The study will adhere to all data protection regulations and all data will be held in accordance with The General Data Protection Regulation (2018).

For peer review only

Clinical data

Clinical data involves baseline demographic data and chronic comorbidities, ICU admission information, severity of illness and acute comorbidities and follow-up data. Clinical data relating to the proposed mechanisms of RV dysfunction will also be collected, specifically regarding ARDS, disordered coagulation, myocardial injury and ventilation (Table 1).

Table 1. Clinical and laboratory data to explore mechanisms of RV dysfunction

MECHANISTIC CATEGORY	CLINICAL AND LABORATORY DATA
**ARDS**	Requirement for prone ventilation
	Murray lung injury score and components
	Requirement for paralysis
	Compliance
	Arterial blood gas analysis
	Referral for extra corporeal membrane oxygenation (ECMO)
**Disordered coagulation**	D-Dimer
	Systemic anti-coagulation (prophylactic/therapeutic)
	Confirmed or suspected pulmonary thromboembolism
	Prothrombin time (PT) / activated partial thromboplastin time (aPTT)
	Platelet count
**Myocardial injury**	LV function (from TTE)
	$ScvO_2$
	Natriuretic peptides (BNP or NT-proBNP)
	Troponin
	Acute coronary syndrome treatment
	Electrocardiogram changes

For peer review only

	Inotropic / vasopressor support
	History of / treatment of arrhythmia
**Ventilation**	Fraction of inspired oxygen (FiO_2)
	Ventilatory mode
	Positive end expiratory pressure
	Peak airway pressure
	Driving pressure
	Respiratory rate

Table 1 Legend Clinical and laboratory data to explore mechanisms of RV dysfunction in each mechanistic category; ARDS, disordered coagulation, myocardial injury and ventilation.

Laboratory data

Laboratory data will be obtained from the local biochemistry and haematology laboratory reporting systems on the day of echocardiography and at follow-up.

ECG data

A 12 lead ECG will be anonymised and transferred electronically to the research team for further analysis. Abnormalities in rate, rhythm, conduction and repolarisation (ST-segment deviation, T-wave inversion) will be classified, blind to other clinical data.

Echocardiography data

A focused echocardiography dataset will be used to answer the primary outcome (Table 2). If available, and the echocardiographer's competency and experience permit, further quantitative measures of RV function will be obtained at this time (Table 3).

Table 2. Focused echocardiography dataset relating to primary outcome

ECHOCARDIOGRAPHY PARAMETER	RESULT
**RV Dilatation**	Yes

For peer review only

	No	
	Unable to assess	
**Septal flattening**	Yes	Systole
		Diastole
		Both
	No	
	Unable to assess	
**RV Function**	Normal	
	RV Dysfunction	
	Unable to assess	
**Free text:** *Comments*		
**LV function**	Normal	
	LV Dysfunction	
	Unable to assess	
**LV Dilatation**	Normal	
	LV Dilatation	
	Unable to assess	
**Free text:** *Comments.*		

Table 2 Legend Basic echocardiography dataset to allow determination of the primary outcome measures.

As part of the dataset, a parasternal short axis (PSAX) and apical 4-chamber (A4C) 'loop' will be recorded and saved. For the A4C loop this will take the form of an RV focused view. For both, the images will be optimised, have ECG monitoring present throughout and the frame rate should be

For peer review only

maximised; both will be recorded for 4 beats.[31] This data will be anonymised and transferred electronically to the research team for further analysis.

Table 3. Detailed echocardiography measures of RV function

**ECHOCARDIOGRAPHY PARAMETER**		**RESULT**
**RV area**	**RV end diastolic area**	cm^2
	**RV end systolic area**	cm^2
	**Fractional area change (FAC).**	%
		Unable to assess
**Free text:** *Image quality, RWMA, McConnell's sign, presence of clot etc.*		
**Triscupid annular plane systolic excursion (TAPSE)**		mm
		Unable to assess
**S' wave velocity at tricuspid annulus (S'Wave)**		cm/s
		Unable to assess
**RV dimensions**	**RVD1**	mm
	**RVD2**	mm
	**RVD3**	mm
		Unable to assess
**TR V_{max}** (to allow calculation of PA systolic pressure)		m/s
		Unable to assess
**Tricuspid regurgitation**		None

For peer review only

			Mild
			Moderate
			Severe
			Unable to assess
**Free text:** *Method of assessing TR (vena contracta, quantitative [EROA, ERV], hepatic vein flow*			
**Right ventricular index of myocardial performance (RIMP) / Tei Index**	**PWD**		
	**Tissue Doppler**		
			Unable to assess
**IVC diameter**	**IVC Diameter**		cm
	**Resp variation /collapse >50%**		Yes
			No
			Unable to assess
**RVOT Acceleration Time**			ms
			Unable to assess
**LV function and size**	**2D**	**LVIDd**	cm
		**LVIDs**	cm
		**LVFS**	%
			Unable to assess
	**Bi-Plane**	**LVEDV**	ml
		**LVESV**	ml
		**LVEF**	%

For peer review only

			Unable to assess
**Free text:** *Comments such as regional wall motion abnormality, valve pathology, etc etc.*			

Table 3. Legend Detailed echocardiography measures of RV function to be undertaken dependent upon the echocardiographer's competency and experience and if image quality allows.

Study Outcomes

Primary outcome

The primary outcome of the study is the prevalence of RV dysfunction in ventilated patients with COVID-19 and its association with 30-day mortality. RV dysfunction will be defined as TTE evidence of RV dilatation along with the presence of septal flattening (in systole, diastole or both). RV dilatation will be determined from the A4C view at end diastole and will be defined as when the RV:LV ratio is >1:1.

Justification for primary outcome

TTE is the mainstay of RV imaging in ICU; it is non-invasive, free from ionising radiation widely available and low cost. The ability to bring the echo machine to the patient bedside means it is the preferred method for diagnosis of RV dysfunction in ICU patients.[32] TTE can determine RV dilatation and can detect eccentric movement of the interventricular septum. The combination of RV dilatation and eccentric septal motion is often referred to as acute cor pulmonale and is a widely reported measure of RV dysfunction in patients with respiratory failure in intensive care.[13, 14, 33-37]

Exploratory outcomes

Exploratory outcome measures largely relate to the association of the proposed aetiological factors, namely ARDS, disordered coagulation, direct myocardial injury and ventilation, to RV dysfunction and 30 day mortality. The association of cardiac biomarkers (troponin T or I and natriuretic peptides (BNP or NT-proBNP)) to the primary outcome measures will also be evaluated.

Saved echo-loops will be analysed centrally at the host institution, blinded and anonymised under the supervision of BSE accredited echocardiographers. In addition to exploring the utility of other echocardiographic parameters such as; eccentricity index and the TAPSE/PA systolic pressure ratio,

For peer review only

2D speckle tracking will be performed for analysis for RV free-wall and global longitudinal strain. Image analysis will be performed on the TOMTEC 2D-Cardiac Performance Analysis platform. Agreement on classification of echocardiographic parameters between critical care echocardiographers at each study site and echocardiographers at the host centre will be explored.

Statistical considerations

All statistical analyses will be performed in conjunction with the Robertson Centre for Biostatistics at the University of Glasgow.

Sample size calculation

Sample size selection is required to be a pragmatic balance of maximising available information versus the prompt delivery of the study and reporting of results to the clinical workforce. Given the number of patients admitted to ICU in Scotland during the early stages of the pandemic, it is realistic to recruit 120-150 patients across participating sites.[5]

Prevalence of RV dysfunction: with a sample size of 120, if the proportion of patients with RV dysfunction was 50%, the 95% confidence interval around the estimated rate would be 40.7% to 59.3%, if the proportion was 25% the 95% confidence interval would be 17.5% to 33.7%. With a sample size of 150 patients, the 95% confidence intervals would be (41.7%, 58.3%) and (18.6%, 33.1%) respectively.

Association with mortality: Table 4 demonstrates the differences in mortality rates that could be detected in a sample size of 120-150 patients at a significance level of 5% and proportions of patients with RV dysfunction of 25% and 50%, assuming an overall mortality rate of 50% (as presented in the recent UK ICNARC report on COVID-19 in critical care).[38]

Table 4. Indicative Power

**Sample Size**	**Power**	**Number *without* RV dysfunction**	**Number *with* RV dysfunction**	**Mortality in patients *without* RV dysfunction**	**Mortality in patients *with* RV dysfunction**	**Odds Ratio**
120	80%	60	60	0.373	0.627	2.83
120	90%	60	60	0.354	0.646	3.33

For peer review only

150	80%	75	75	0.386	0.614	2.53
150	90%	75	75	0.369	0.631	2.92
120	80%	90	30	0.427	0.719	3.43
120	90%	90	30	0.417	0.749	4.17
150	80%	112	38	0.435	0.695	2.96
150	90%	112	38	0.426	0.722	3.50

Table 4. Legend Differences in mortality rates that could be detected at a 5% significance level for sample sizes of 120 and 150, and proportions of patients with RV dysfunction of 25% and 50%, assuming an overall mortality rate of 50%.

Data analysis

Primary outcome

The proportion of ventilated patients with COVID-19 who have RV dysfunction will be determined, with a 95% confidence interval (Clopper-Pearson method). The association of RV dysfunction with 30-day mortality will then be analysed using logistic regression analysis predicting 30-day ICU mortality from presence or absence of RV dysfunction, adjusting for patient demographics, phase of disease and baseline severity of illness.

Exploratory outcomes

To assess the role of potential aetiological factors (ARDS, disordered coagulation, direct myocardial injury and ventilation) for RV dysfunction, the relation of each measure of the aetiological factors to the outcomes will be analysed in the same way as for the primary outcome. A more in-depth analysis will include carrying out a principal component analysis for all measures within each factor, in order to create one single measure (the first principal component) representing each factor. The relation of these to the outcomes will be assessed in regression analyses analogously to the ones described above. This will be done for each factor separately and also in combination. Other approaches, e.g. the least absolute shrinkage and selection operator (LASSO), for determining the role of the individual measures of all factors may be explored.[39]

The association of cardiac biomarkers with RV dysfunction will be analysed using logistic regression analysis predicting presence or absence of RV dysfunction from the biomarker level, adjusting for patient demographics, phase of disease and baseline severity. Association of the biomarkers with

For peer review only

30-day mortality will be analysed in a similar manner. To assess whether any association differs in the presence or absence of RV dysfunction, RV dysfunction and its interaction with the biomarker will be added to the model.

Patient and public involvement statement

Due to the nature of the current COVID-19 pandemic and the subsequent urgency to establish this study there was no patient or public involvement. Patients were not invited to comment on the study design and were not consulted to develop patient relevant outcomes or interpret the results. Patients were not invited to contribute to the writing or editing of this document for readability or accuracy.

For peer review only

ETHICS AND DISSEMINATION

Ethical approval was granted by the Scotland A Research Ethics Committee (REC reference 20/SS/0059) on 05/06/2020.

The safety profile of all research interventions; TTE, collection of clinical information and blood specimens, is well established and therefore adverse event reporting is not applicable.

Given the nature of the current global pandemic we will endeavor to publish the prevalence of RV dysfunction in COVID-19 as soon as possible on completion of the study. Once the thirty-day follow-up period is complete, the primary outcome will be published along with all available exploratory analyses. We envisage sharing findings with the critical care community at an early stage through a series of webinars.

For peer review only

REFERENCES

1. Zhu N, Zhang D, Wang W, et al. A Novel Coronavirus from Patients with Pneumonia in China, 2019. The New England journal of medicine 2020;382(8):727-33 doi: 10.1056/NEJMoa2001017[published Online First: Epub Date]|.

2. Wu Z, McGoogan JM. Characteristics of and Important Lessons From the Coronavirus Disease 2019 (COVID-19) Outbreak in China: Summary of a Report of 72314 Cases From the Chinese Center for Disease Control and Prevention. Jama 2020 doi: 10.1001/jama.2020.2648[published Online First: Epub Date]|.

3. World Health Organisation. Coronavirus disease (COVID-19) Situation Report. Secondary Coronavirus disease (COVID-19) Situation Report 28/09/2020. https://www.who.int/docs/default-source/coronaviruse/situation-reports/20200928-weekly-epi-update.pdf?sfvrsn=9e354665_6.

4. Intensive Care National Audit & Research Centre (ICNARC). ICNARC report on COVID-19 in critical care 25 September 2020 Secondary ICNARC report on COVID-19 in critical care 25 September 2020 2020. https://www.icnarc.org/Our-Audit/Audits/Cmp/Reports.

5. Scottish Intensive Care Society Audit Group (SICSAG). Scottish Intensive Care Society Audit Group report on COVID-19 20th June 2020. Secondary Scottish Intensive Care Society Audit Group report on COVID-19 20th June 2020 08/07/2020. https://beta.isdscotland.org/find-publications-and-data/population-health/covid-19/scottish-intensive-care-society-audit-group-report-on-covid-19/.

6. Gattinoni L, Chiumello D, Rossi S. COVID-19 pneumonia: ARDS or not? Critical Care 2020;24(1):154 doi: 10.1186/s13054-020-02880-z[published Online First: Epub Date]|.

7. Fried JA, Ramasubbu K, Bhatt R, et al. The Variety of Cardiovascular Presentations of COVID-19. Circulation;0(0) doi: doi:10.1161/CIRCULATIONAHA.120.047164[published Online First: Epub Date]|.

8. Ruan Q, Yang K, Wang W, et al. Clinical predictors of mortality due to COVID-19 based on an analysis of data of 150 patients from Wuhan, China. Intensive Care Med 2020 doi: 10.1007/s00134-020-05991-x[published Online First: Epub Date]|.

9. Szekely Y, Lichter Y, Taieb P, et al. The Spectrum of Cardiac Manifestations in Coronavirus Disease 2019 (COVID-19) - a Systematic Echocardiographic Study. Circulation 2020 doi: 10.1161/CIRCULATIONAHA.120.047971[published Online First: Epub Date]|.

For peer review only

10. Lahm T, Douglas IS, Archer SL, et al. Assessment of Right Ventricular Function in the Research Setting: Knowledge Gaps and Pathways Forward. An Official American Thoracic Society Research Statement. Am J Respir Crit Care Med 2018;198(4):e15-e43 doi: 10.1164/rccm.201806-1160ST[published Online First: Epub Date]|.

11. Grignola JC, Domingo E. Acute Right Ventricular Dysfunction in Intensive Care Unit. BioMed research international 2017;2017:8217105 doi: 10.1155/2017/8217105[published Online First: Epub Date]|.

12. Zochios V, Parhar K, Vieillard-Baron A. Protecting the Right Ventricle in ARDS: The Role of Prone Ventilation. Journal of Cardiothoracic and Vascular Anesthesia 2018;32(5):2248-51 doi: https://doi.org/10.1053/j.jvca.2018.01.007[published Online First: Epub Date]|.

13. Boissier F, Katsahian S, Razazi K, et al. Prevalence and prognosis of cor pulmonale during protective ventilation for acute respiratory distress syndrome. Intensive care medicine 2013;39(10):1725-33 doi: 10.1007/s00134-013-2941-9[published Online First: Epub Date]|.

14. Mekontso Dessap A, Boissier F, Charron C, et al. Acute cor pulmonale during protective ventilation for acute respiratory distress syndrome: prevalence, predictors, and clinical impact. Intensive Care Med 2016;42(5):862-70 doi: 10.1007/s00134-015-4141-2[published Online First: Epub Date]|.

15. Shah TG, Wadia SK, Kovach J, et al. Echocardiographic parameters of right ventricular function predict mortality in acute respiratory distress syndrome: a pilot study. Pulm. Circ. 2016;6(2):155-60 doi: 10.1086/685549[published Online First: Epub Date]|.

16. Li Y, Li H, Zhu S, et al. Prognostic Value of Right Ventricular Longitudinal Strain in Patients with COVID-19. JACC: Cardiovascular Imaging 2020:3423 doi: 10.1016/j.jcmg.2020.04.014[published Online First: Epub Date]|.

17. Argulian E, Sud K, Vogel B, et al. Right Ventricular Dilation in Hospitalized Patients with COVID-19 Infection. JACC. Cardiovascular imaging 2020 doi: 10.1016/j.jcmg.2020.05.010[published Online First: Epub Date]|.

18. Vieillard-Baron A, Matthay M, Teboul JL, et al. Experts' opinion on management of hemodynamics in ARDS patients: focus on the effects of mechanical ventilation. Intensive care medicine 2016;42(5):739-49 doi: 10.1007/s00134-016-4326-3[published Online First: Epub Date]|.

For peer review only

19. Paternot A, Repesse X, Vieillard-Baron A. Rationale and Description of Right Ventricle-Protective Ventilation in ARDS. Respiratory care 2016;61(10):1391-6 doi: 10.4187/respcare.04943[published Online First: Epub Date]|.

20. British Thoracic S. BTS Guidance on Venous Thromboembolic Disease in patients with COVID-19. Secondary BTS Guidance on Venous Thromboembolic Disease in patients with COVID-19 2020. file:///C:/Users/mccal/Downloads/BTS%20Guidance%20on%20Venous%20Thromboembolic%20Disease%20in%20patients%20with%20COVID-19.pdf.

21. Zhou F, Yu T, Du R, et al. Clinical course and risk factors for mortality of adult inpatients with COVID-19 in Wuhan, China: a retrospective cohort study. The Lancet 2020;395(10229):1054-62 doi: 10.1016/S0140-6736(20)30566-3[published Online First: Epub Date]|.

22. Liu PP, Blet A, Smyth D, et al. The Science Underlying COVID-19: Implications for the Cardiovascular System. Circulation;0(0) doi: doi:10.1161/CIRCULATIONAHA.120.047549[published Online First: Epub Date]|.

23. Klok FA, Kruip MJHA, van der Meer NJM, et al. Incidence of thrombotic complications in critically ill ICU patients with COVID-19. Thrombosis Research 2020 doi: https://doi.org/10.1016/j.thromres.2020.04.013[published Online First: Epub Date]|.

24. Fox SE, Akmatbekov A, Harbert JL, et al. Pulmonary and Cardiac Pathology in Covid-19: The First Autopsy Series from New Orleans. medRxiv 2020:2020.04.06.20050575 doi: 10.1101/2020.04.06.20050575[published Online First: Epub Date]|.

25. Acute Respiratory Distress Syndrome N, Brower RG, Matthay MA, et al. Ventilation with lower tidal volumes as compared with traditional tidal volumes for acute lung injury and the acute respiratory distress syndrome. The New England journal of medicine 2000;342(18):1301-8 doi: 10.1056/NEJM200005043421801[published Online First: Epub Date]|.

26. Inciardi RM, Lupi L, Zaccone G, et al. Cardiac Involvement in a Patient With Coronavirus Disease 2019 (COVID-19). JAMA Cardiology 2020 doi: 10.1001/jamacardio.2020.1096[published Online First: Epub Date]|.

27. Augoustides JG. Cardiovascular Consequences and Considerations of Coronavirus Infection – perspectives for the cardiothoracic anesthesiologist and intensivist during the coronavirus crisis. Journal of cardiothoracic and vascular anesthesia 2020 doi: https://doi.org/10.1053/j.jvca.2020.04.001[published Online First: Epub Date]|.

For peer review only

28. Murphy E, Shelley B. Clinical presentation and management of right ventricular dysfunction. BJA Education 2019;19(6):183-90 doi: 10.1016/j.bjae.2019.02.004[published Online First: Epub Date]|.

29. Vieillard-Baron A, Charron C, Caille V, et al. Prone positioning unloads the right ventricle in severe ARDS. Chest 2007;132(5):1440-6 doi: 10.1378/chest.07-1013[published Online First: Epub Date]|.

30. Intensive Care Society. FICE Accreditation Pack. Secondary FICE Accreditation Pack 2017.

31. Zaidi A, Knight DS, Augustine DX, et al. Echocardiographic assessment of the right heart in adults: a practical guideline from the British Society of Echocardiography. 2020;7(1):G19 doi: 10.1530/erp-19-0051[published Online First: Epub Date]|.

32. Vieillard-Baron A, Naeije R, Haddad F, et al. Diagnostic workup, etiologies and management of acute right ventricle failure : A state-of-the-art paper. Intensive care medicine 2018;44(6):774-90 doi: 10.1007/s00134-018-5172-2[published Online First: Epub Date]|.

33. Repessé X, Charron C, Vieillard-Baron A. Acute Cor Pulmonale in ARDS: Rationale for Protecting the Right Ventricle. Chest 2015;147(1):259-65 doi: https://doi.org/10.1378/chest.14-0877[published Online First: Epub Date]|.

34. Vieillard-Baron A, Prin S, Chergui K. Echo–Doppler demonstration of acute cor pulmonale at the bedside in the medical intensive care unit. Am J Respir Crit Care Med. 2002;166 doi: 10.1164/rccm.200202-146CC[published Online First: Epub Date]|.

35. Vieillard-Baron A, Schmitt JM, Augarde R. Acute cor pulmonale in acute respiratory distress syndrome submitted to protective ventilation: incidence, clinical implications, and prognosis. Crit Care Med. 2001;29 doi: 10.1097/00003246-200108000-00009[published Online First: Epub Date]|.

36. Krishnan S, Schmidt GA. Acute right ventricular dysfunction: real-time management with echocardiography. Chest 2015;147(3):835-46 doi: 10.1378/chest.14-1335[published Online First: Epub Date]|.

37. Huang SJ, Nalos M, Smith L, et al. The use of echocardiographic indices in defining and assessing right ventricular systolic function in critical care research. Intensive Care Medicine 2018;44(6):868-83 doi: 10.1007/s00134-018-5211-z[published Online First: Epub Date]|.

38. Intensive Care National Audit & Research Centre (ICNARC). ICNARC report on COVID-19 in critical care. 24th April 2020. Secondary ICNARC report on COVID-19 in critical care. 24th April 2020 2020.

For peer review only

https://www.icnarc.org/DataServices/Attachments/Download/c5a62b13-6486-ea11-9125-00505601089b.

39. Tibshirani R. Regression Shrinkage and Selection via the Lasso. Journal of the Royal Statistical Society. Series B (Methodological) 1996;58(1):267-88

For peer review only

AUTHORS' CONTRIBUTIONS

BS and PM conceived of the study and BS is the grant holder. MG and CB assisted in the initial study design. JW wrote the patient documentation and developed the case report forms and online data collection database. C-MM provided guidance on sample size requirements and statistical analysis and will be overseeing the statistical analyses. All authors contributed to refinement of the study protocol and approved the final manuscript.

FUNDING STATEMENT

This work was supported by Medical Research Scotland, grant number CVG-1730-2020. Professor Colin Berry is supported by British Heart Foundation Centre of Research Excellence grant (reference number RE/18/6/34217).

COMPETING INTERESTS STATEMENT

Dr. Shelley reports personal fees from Janssen-Cilag Ltd, outside the submitted work.

For peer review only
